# Indications for an early invasive strategy in NSTE-ACS patients

**DOI:** 10.1007/s12471-019-01337-5

**Published:** 2019-11-06

**Authors:** B. Zwart, J. M. ten Berg, A. W. van ’t Hof, P. A. L. Tonino, Y. Appelman, A. H. Liem, F. Arslan, J. Waltenberger, J. W. Jukema, R. J. de Winter, P. Damman

**Affiliations:** 1 Department of Cardiology, Catharina ziekenhuis, Eindhoven, The Netherlands; 2 Department of Cardiology, St. Antonius ziekenhuis, Nieuwegein, The Netherlands; 3 Department of Cardiology, Maastricht University Medical Center (UMC)+, Maastricht, The Netherlands; 4 Department of Cardiology, Amsterdam University Medical Center, Location VU University Medical Center, Amsterdam, The Netherlands; 5 Department of Cardiology, Franciscus Gasthuis, Rotterdam, The Netherlands; 6 Department of Cardiovascular Medicine, Medical Faculty, University of Münster, Münster, Germany; 7 Department of Cardiology, Leiden University Medical Center, Leiden, The Netherlands; 8 Department of Cardiology, Amsterdam University Medical Center, location AMC, University of Amsterdam, Amsterdam, The Netherlands; 9 Department of Cardiology, Radboud University Medical Center, Nijmegen, The Netherlands

**Keywords:** Acute coronary syndrome, Coronary angiography, Timing

## Abstract

An early invasive strategy in patients who have acute coronary syndrome without ST-elevation (NSTE-ACS) can improve clinical outcome in high-risk subgroups. According to the current guidelines of the European Society of Cardiology (ESC), the majority of NSTE-ACS patients are classified as “high-risk”. We propose to prioritise patients with a global registry of acute coronary events (GRACE) risk score >140 over patients with isolated troponin rise or electrocardiographic changes and a GRACE risk score <140. We also acknowledge that same-day transfer for all patients at a high risk is not necessary in the Netherlands since the majority of Dutch cardiology departments are equipped with a catheterisation laboratory where diagnostic coronary angiography is routinely performed in NSTE-ACS patients. Therefore, same-day transfer should be restricted to *true* high-risk patients (in addition to those NSTE-ACS patients with very high-risk (VHR) criteria) in centres without coronary angiography capabilities.

## Introduction

**Fig. 1:**
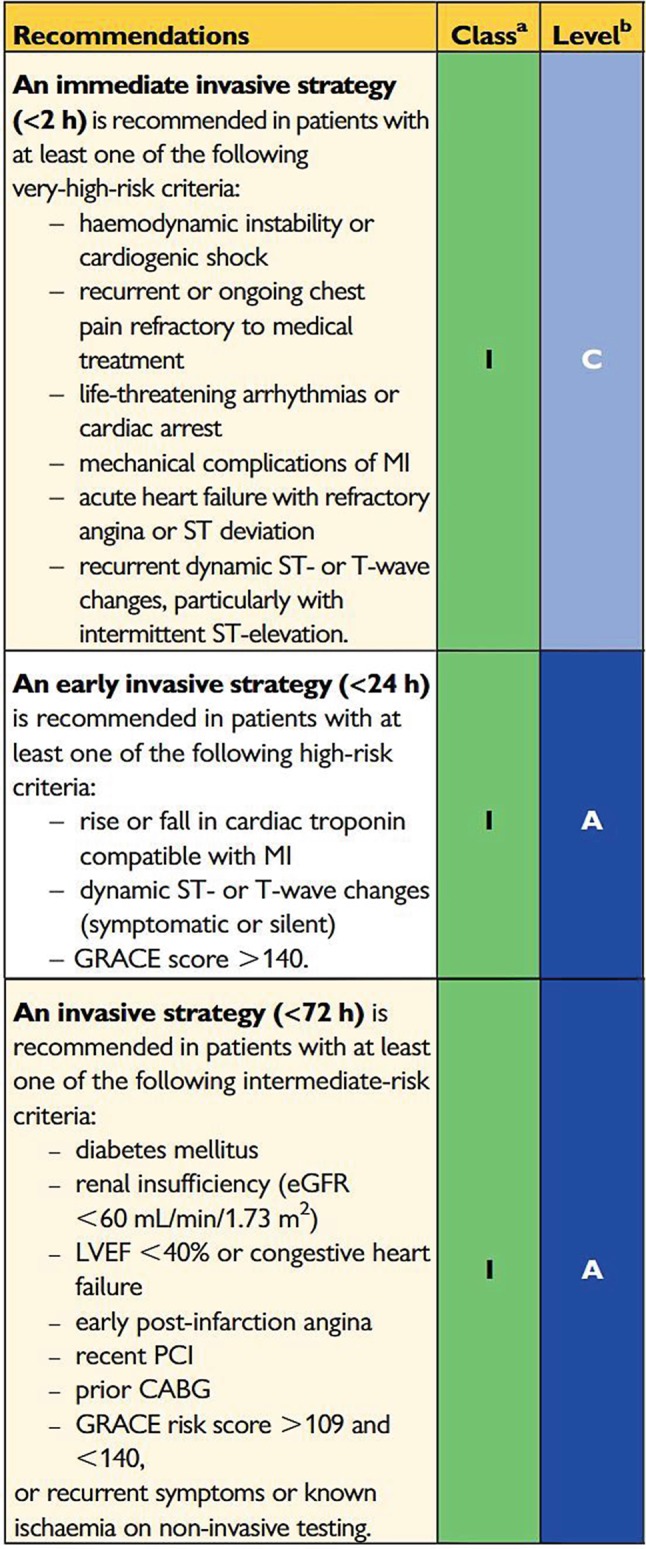
Selection of the 2015 ESC guidelines’ recommendations for invasive coronary angiography and revascularisation in NSTE-ACS. (^a^Class of recommendation, ^b^Level of evidence. *NSTE-ACS* non-ST-elevation acute coronary syndromes, *MI* myocardial infarction, *GRACE* global registry of acute coronary events, *eGFR* estimated glomerular filtration rate, *LVEF* left ventricular ejection fraction, *PCI* percutaneous coronary intervention, *CABG* coronary artery bypass grafting)

Acute coronary syndrome without ST-elevation (NSTE-ACS) represents a broad spectrum of diagnoses, ranging from unstable angina to myocardial infarction with considerable myocardial damage. A routine invasive strategy (coronary angiography within 24–72 h) has been shown to improve clinical outcomes when compared with a selectively invasive strategy (coronary angiography in case of refractory angina and/or inducible ischaemia by non-invasive stress testing [1]). Therefore, the 2015 European Society of Cardiology (ESC) NSTE-ACS guidelines and the 2018 ESC Myocardial revascularisation guidelines endorse an invasive strategy in moderate-risk to high-risk patients, while highlighting the role of risk stratification in the decision process [1, 2]. The timing of an invasive strategy should be based on individual patient risk factors, such as haemodynamic instability, refractory symptoms or clinical characteristics such as diabetes or renal failure (Fig. 1). The current ESC guidelines advise an immediate invasive strategy in patients with ‘very-high characteristics’, such as haemodynamic instability or refractory angina (Fig. 1). An early (<24 h) invasive strategy is recommended in patients with a global registry of acute coronary events (GRACE) risk score >140, dynamic ST-segment and T‑wave (ST-T) changes or a typical rise-and-fall in cardiac troponin. Moreover, the ESC guidelines recommend a “same-day transfer” to a centre for percutaneous coronary intervention (PCI) in these high-risk patients.

The current paper discusses the evidence and limitations of these recommendations for an early invasive strategy, as well as specific comments on the Dutch situation.

## Background

The Timing of Intervention in Acute Coronary Syndromes (TIMACS) trial, which was published in 2009, was the largest trial thus far randomising a total of 3,031 patients to coronary angiography <24 h versus >36 h after randomisation [3]. The study was not able to demonstrate an overall benefit of an invasive strategy with regard to the primary composite endpoint of death, myocardial infarction or stroke at six months. However, in a prespecified analysis of high-risk patients with a GRACE risk score >140 (one third of patients), an early invasive strategy did confer a significant reduction of the primary endpoint (hazard ratio [HR] 0.65, confidence interval [CI] 0.48–0.89,* p* = 0.006). Other randomised controlled trials showed variable results and were not able to demonstrate a consistent benefit of an early invasive strategy [4–9].

Several subsequent meta-analyses have shown a benefit in terms of recurrent or refractory ischaemia and length of hospital stay associated with an early invasive strategy compared with a delayed invasive strategy [10–12]. Results of an early strategy on mortality and non-fatal myocardial infarction are less consistent in studies [10–14].

Li et al. conducted a systematic review and meta-analysis and found a significant reduction in both refractory ischaemia and mortality associated with early invasive strategy versus delayed invasive strategy, but no reduction in myocardial infarction [15]. Another meta-analysis of randomised controlled trials based on individual patient data by Jobs et al. did not demonstrate an overall mortality benefit associated with an early invasive strategy [16]. However, in a prespecified analysis in patients with elevated biomarkers at baseline, a mortality benefit was found. In addition, in subgroups of patients with diabetes, age >75 years and a GRACE risk score >140 a mortality benefit was observed, although tests for interaction were inconclusive. The effect observed in this meta-analysis was still largely driven by the results of the TIMACS trial, which, together with the ELISA‑3, made up 65% of all patients. Furthermore, this meta-analysis included a substantial proportion of ‘older’ studies (range 2000–2016), during which time frame substantial developments were achieved with respect to pharmacotherapy and the availability of high-sensitive troponin essays.

Very recently, the Very Early veRsus Deferred Invasive evaluation using Computerized Tomography (VERDICT) trial was published [17]. Patients with a clinical suspicion of NSTE-ACS were included, based on either new electrocardiographic (ECG) changes compatible with myocardial ischaemia, or positive cardiac troponin. The primary endpoint of all-cause death, non-fatal recurrent myocardial infarction, hospital admission for refractory myocardial ischaemia or hospital admission for heart failure did not differ between the early invasive group (coronary angiography with possible revascularisation <12 h) versus the delayed invasive group (angiography with possible revascularisation in 48–72 h). However, among patients with a GRACE risk score >140, a reduction of the primary endpoint was observed (HR 0.81, 95% CI 0.67–1.01, *p*-value for interaction = 0.023). This result is consistent with the TIMACS trial, however, it is not known which separate endpoint drove this outcome difference.

Data regarding possible sex differences in early invasive strategy are scarce. Several studies suggest that an early invasive strategy is beneficial in women with NSTE-ACS, but not in low-risk women (i.e. negative biomarkers) [18, 19]. However, another study suggested caution in extrapolating the results from men to women in the context of choosing a therapeutic strategy in ACS [20].

## Recommendations

In general, an early invasive strategy in selected patients seems to result in better outcomes than a delayed invasive strategy. In selected high-risk subgroups, a mortality reduction is suggested. Therefore, it is vital to identify those patients at high risk who might benefit from such a strategy. Not surprisingly, clinical characteristics in the GRACE risk calculator such as cardiac arrest, signs of heart failure and haemodynamic instability have the largest impact.

The “high-risk” criteria as listed in the present ESC guidelines seem to overestimate the risk. Apart from a GRACE risk score >140, patients with isolated troponin rise or ST‑T changes classify as “high-risk” as well. Arguably, the risk of these latter two groups is lower than the risk of patients with a GRACE risk score >140. This is in part a result of the availability of more sensitive troponin tests, whereas most trials were performed before these high-sensitivity troponin assays became the clinical standard in Europe. It is not clear to what extent these highly sensitive assays might impact the applicability of the currently available study results, but it is possible that a substantial number of NSTE-ACS patients in current practice would previously have been classified as having unstable angina.

In our opinion, the strongest evidence for an early invasive strategy is in patients with a GRACE risk score >140. For patients with positive cardiac markers or ST‑T changes but with a GRACE risk score <140 (who comprise the majority of NSTE-ACS patients in contemporary practice) no compelling evidence exists that such patients should undergo coronary angiography within 24 h. Importantly, the VERDICT trial, which was a large trial including such patients with either positive biomarkers or ST‑T changes did not show an overall benefit of an invasive strategy. This study was published after the introduction of the 2015 and 2018 ESC guidelines.

Importantly, as the current guidelines criteria identify the majority of contemporary NSTE-ACS as “high-risk”, there is a risk of losing sight of the *true* high-risk patients. This may lead to a policy of not prioritising the *true* high-risk patients undergoing invasive coronary angiography within 24 h. As it is key to identify the *true* high-risk patients and based on the available evidence, we recommend prioritising patients with a GRACE risk score >140 (including clinical parameters such as heart failure, arrest and haemodynamic instability) over patients with isolated positive troponin or isolated ST‑T changes.

## Transfer to a PCI centre

The 2015 NSTE-ACS guidelines recommend a ‘same-day transfer’ to a PCI centre for high-risk patients based on the aforementioned criteria. For the Dutch situation, this is—in our opinion—not a necessity, as discussed before in a position paper by members of the Dutch Society for Cardiology Acute Coronary Syndrome working group [21, 22]. The evidence for an invasive strategy stems primarily from studies investigating the timing of coronary angiography—and not timing of revascularisation necessarily.

The Dutch situation is markedly different from that in many other European countries since the majority of Dutch cardiology departments are equipped with a catheterisation laboratory where diagnostic coronary angiography is routinely performed in NSTE-ACS patients. After diagnostic angiography, patients are discussed in a heart team and based on patient characteristics and coronary anatomy a prioritisation can be made for the transfer to an interventional centre for those suitable for PCI or coronary artery bypass grafting (CABG).

When coronary angiography is not available in the admitting centre or is not possible for other reasons, transfer within 24 h to a PCI centre may be considered. However, urgent (same-day) transfer should be restricted to high-risk patients with a GRACE risk score >140 or with very-high-risk criteria as mentioned in the ESC guidelines (Fig. 1). We advocate regional arrangements to be made between hospitals without 24/7 angiography capacities and local PCI centres.

## Recommendations

- Identification of high-risk NSTE-ACS patients is important and can improve outcomes if coronary angiography is performed within 24 h in order to determine an appropriate revascularisation strategy.
- According to the current ESC guidelines, the majority of NSTE-ACS patients are classified as ‘high-risk’. Based on currently available evidence, we propose to prioritise patients with a GRACE risk score >140 over patients with isolated troponin rise or ECG changes and a GRACE risk score <140.
- In centres without coronary angiography capabilities, same-day transfer to a PCI centre should be considered in *true* high risk patients with a GRACE risk score >140 or other unstable characteristics
